# Potential Role of SLC5A8 Expression in the Etiology of Subacute Ruminal Acidosis

**DOI:** 10.3389/fvets.2020.00394

**Published:** 2020-07-30

**Authors:** Chenxu Zhao, Gerd Bobe, Yazhou Wang, Xinyue Zhang, Zhibo Zhao, Shiqi Zhang, Guoquan Sun, Xue Yuan, Xinwei Li, Guowen Liu

**Affiliations:** ^1^Key Laboratory of Zoonosis Research, Ministry of Education, College of Veterinary Medicine, Jilin University, Changchun, China; ^2^Department of Animal Sciences, Linus Pauling Institute, Oregon State University, Corvallis, OR, United States; ^3^College of Animal Science and Technology, Inner Mongolia National University, Tongliao, China

**Keywords:** subacute ruminal acidosis, solute-linked carrier 5a8, short chain fatty acids, rumen epithelium, dairy cows

## Abstract

Rumen fluid of cows with subacute ruminal acidosis (SARA) has high concentrations of short chain fatty acids (SCFA). However, the mechanism of SCFA accumulation is unknown. The solute-linked carrier 5a8 (SLC5A8) plays a key role in the transportation and absorption of SCFA in the intestinal epithelium. The objective of the current study was to investigate (1) SLC5A8 gene and protein expression in various parts of the bovine gastrointestinal tract, (2) the effect of SCFA on SLC5A8 expression in rumen epithelial cells, and (3) SLC5A8 gene and protein expression in SARA and healthy cows. A total of 10 dairy cows, 84 ± 26 days in milk and in their second to fourth parity were allocated to control (*n* = 5) and SARA groups (*n* = 5). Three cows from the control group and three calves (1-day-old, female, 45–50 kg, healthy, fasting) were chosen to collect a total of 10 sections of digestive tract, from rumen to rectum, and then bovine ruminal epithelial cells were isolated from the three calves. Gene and protein expression of SLC5A8 was detected in all tested regions of the gastrointestinal tract in calves and adult cows by Western blot and quantitative real-time PCR and were both highest in the rumen. Gene and protein expression of SLC5A8 was more than 50% lower in the rumen epithelium of SARA vs. control cows and was partly restored after therapy of SARA cows. Compared with SCFA concentrations typical for control cows (60 m*M* acetate, 30 m*M* propionate, and 20 m*M* butyrate), gene and protein expression of SLC5A8 in rumen epithelium was lower at elevated SCFA concentrations typical for SARA cows (90 m*M* acetate, 40 m*M* propionate, and 30 m*M* butyrate), specifically for elevated concentrations of propionate or butyrate in contrast to elevated concentrations of acetate increased gene and protein expression of SLC5A8 in rumen epithelium. In conclusion, the elevated concentrations of propionate and butyrate inhibit ruminal absorption of SCFA via downregulation of SLC5A8 in SARA cows; the expression of SLC5A8 plays an important role in the etiology of SARA.

## Introduction

Subacute ruminal acidosis (SARA) is a common, difficult to diagnose, and costly digestive disorder in dairy cows. It is commonly diagnosed by an occurrence of ruminal pH < 5.6 for >3 h/day (1) and the occurrence of clinical signs, such as intermittent diarrhea, dehydration, poor body condition, depression, decreased rumen motility, laminitis, or decreased milk production (2, 3). The incidence rate of SARA is between 11 and 26% in dairy cows (4–6), and the estimated cost is $216 per case (7).

Compared with healthy cows, cows with SARA have elevated concentrations of short chain fatty acids (SCFA) in the rumen (8, 9), which could be due to greater SCFA production, decreased SCFA absorption across the rumen epithelium (3, 8), or both. Intestinal SCFA absorption in rats and humans is mediated by solute-linked carrier 5a8 (SLC5A8) (10), a low-affinity/high-capacity SCFA^−^/bicarbonate antiporter (11), whose affinity in zebrafish in descending order of greatest affinity is butyrate > propionate > acetate (12). In addition, SLC5A8 has high protein homology among rat, human, and cattle (from NCBI). Given the accumulation of SCFA in the rumen during SARA, we, therefore, hypothesized that SLC5A8 may play a role in the etiology of SARA.

To determine the potential role of SLC5A8 in the etiology of SARA, our first objective was to determine the currently unknown distribution of protein and gene expression SLC5A8 across the bovine gastrointestinal tract. Our second objective was to evaluate whether gene and protein expression of SLC5A8 in rumen epithelial cells differed between SARA and healthy cows and in SARA cows before and after successful therapy. Our third objective was to evaluate whether accumulation of SCFA individually and as a group alters gene and protein expression of SLC5A8 expression in the rumen epithelium in a manner that could induce SARA.

## Materials and Methods

### Animals and Diets

Animal experiments in this study were performed in accordance with the Guiding Principles of Animals adopted by the Chinese Association for Laboratory Animal Sciences. All of the animal procedures were approved by the Institutional Animal Care and Use Committee of Jilin University, China.

A case-control study was conducted to compare SLC5A8 expression between healthy cows and cows with SARA before and after therapeutic treatment for SARA. To have a statistically significant difference in the relative rumen epithelial SLC5A8 level between healthy and SARA cows, we ran a calculation for sample size with *P* at 0.01 and power at 95%. We assumed that the standard deviation of relative rumen epithelial SLC5A8 levels was 0.3 and would like to see a 1-fold difference in relative SLC5A8 abundance between healthy cows and cows with SARA (https://www.bu.edu/researchsupport/compliance/animal-care/working-with-animals/research/sample-size-calculations-iacuc/). Three or more animals should be included in each group. Hence, five lactating healthy Holstein cows were matched by parity and lactation stage with five cows with SARA. Cows were selected from a 3,000-cow ecology dairy farm located in Suihua City, Heilongjiang Province, China. To select cows with SARA, first, two experienced independent veterinarians identified cows with two or more of the following clinical presentations: intermittent diarrhea, dehydration, poor body condition, depression, decreased rumen motility, laminitis, or decreased milk production (2). Twenty-one cows were identified. Second, rumen pH was measured for each of these identified cows a total of six times at 30 min intervals starting from 6 h after feeding, and the cows whose pH were <5.6 at each measurement were considered to have SARA. A total of seven cows met this requirement. The five cows whose average ruminal pH were the lowest were selected as the SARA group. Ten healthy asymptomatic cows were preselected at random by certified veterinarians, and their rumen pH was measured using the same method as that for measurement of pH for the SARA cows. The five cows whose ruminal pH > 5.8 and average pH were the highest were selected as control group. The weight of cows was measured daily for 2 consecutive days after the selection of SARA and control group by platform scale (<1,000 kg; #SCS; Dingtuo, Shanghai, China). Cows were 84 ± 26 days in milk (mean ± standard deviation, *SD*) and were in their 2–4 parity. Healthy cows had an average milk yield of 31.6 ± 2.9 kg/d and an average body weight of 580 ± 44 kg. SARA cows had an average milk yield of 27.5 ± 1.5 kg/d and an average body weight of 570 ± 39 kg. In addition, three Holstein calves (1-day-old, female, 45–50 kg, healthy, fasting) were selected for subsequent experiments.

Cows were fed *ad libitum* a TMR (Table 1) twice per day at 7:00 and 16:30, which met the nutritional requirements of the animals (NRC, 2001). The average dry matter intake was 20–22 kg/d per cow. Cows were milked twice per day at 5:30 and 15:00. After the rumen epithelial samples were collected, SARA cows recovered for 1 week from rumen incision and then were treated for 3 weeks daily with 1,310 g of a combination of buffers (Jiaxin Animal Pharmaceutical Ltd., Tangshan, China) added to the TMR according to Downer and Cummings (13). Table 2 lists the doses of various compounds used as buffers.

**Table 1:** Feed and nutrient composition of diet.

**Feed Composition**	**Percent of Diet Dry Matter**
Corn silage	40.00
Corn	35.00
Wheat bran	8.00
Soybean meal	5.00
Sunflower	8.00
NaCl	1.00
Premix^a^	1.80
NaHCO_3_	1.20
Total	100.00
Nutrient composition	
NE_L_ (MJ/Kg)	6.71
CP	18.15
NDF	30.72
ADF	20.10
NFC^b^	38.40
Crude fat	5.03
Ash	7.70
Ca	1.32
P	0.41

a *The premix provided the following per kg of diets: vitamin A 190,000 IU, vitamin D 68,000 IU, vitamin E 1310 IU, Fe 2,500 mg, Cu 600 mg, Zn 2,400 mg, Mn 1,100 mg, I 8 mg, Co 7 mg*.

b *NFC =100—(NDF% + CP % + Crude fat % + Ash %), Non-fiber carbohydrate*.

*NE_L_, Net energy for lactation; CP, Crude protein; NDF, Neutral detergent fiber; ADF, Acid detergent fiber. The diet composition and nutrient analysis were derived using Resource Formulator software version Refs 3000 (Beijing, China)*.

**Table 2:** The doses of various buffers added to the feed rations.

**Product**	**Amount (g/day)**
Sodium bicarbonate	200
Sodium sesquicarbonate	180
Magnesium oxide	60
Sodium bentonite	350
Calcium carbonate	160
Potassium carbonate	360

### Rumen Fluid Collection and SCFA Analysis

Rumen fluid collection from healthy cows and cows with SARA before and after therapeutic treatment started 6 h after the morning feeding because this time coincides with the predicted daily nadir in rumen pH in TMR-fed herds (14). The collection of rumen fluid was conducted every 30 min for 3 h using a needle from a 14-gauge, 80 mm-long, intravenous catheter and a 20-mL syringe. The rumen fluid pH was measured immediately using a calibrated electronic pH meter (Hanna Instruments pH 210 Microprocessor pH meter, Woonsocket, USA). Twenty milliliters of rumen fluid was then transferred into a 50-ml centrifuge tube (430828; Corning, NY, USA) containing 1 mL of 70 m*M* mercuric chloride (7487-94-7; TongJie Chemical reagent Co., Ltd., Shanxi, China) to prevent further fermentation. Samples were stored on dry ice for 60 min until they were transported to the laboratory (5). Ruminal contents were filtered through four layers of cheesecloth. Rumen fluid samples were centrifuged at 2,500 × g for 15 min by centrifuge (Model X-30R, Beckman Coulter, Inc., USA). The supernatant was frozen at −80°C. The supernatant was used to determine the concentrations of SCFA (acetate, propionate, butyrate, and isobutyrate) and lactate (*D-* and *L-*) by gas chromatography (15). Ruminal fluid was injected by a sampler (model 8200, Varian, Walnut Creek, CA) into a Stabilwax-DA column (30 m × 0.53 mm i.d. × 0.5 μm film) on a gas chromatographer (Model 3400 Star, Varian, Walnut Creek, CA). The samples were run at a split ratio of 20:1 with a column temperature of 90 to 170°C with an increase of 10°C/min to hold 2 min. The injector and detector temperatures were 170 and 190°C, respectively. The chromatographic peak integration was performed using Galaxie Software (Varian).

### Tissue Collection

To compare SLC5A8 expression in ruminal epithelium between healthy cows and cows with SARA before and after SARA therapeutic treatment, tissue samples of rumen epithelia were obtained by making an incision in the left abdomen through surgical procedures by experienced veterinarians: First, the position of the left para-lumbar fossa to dorsal midline was sheared and disinfected by 5% iodine tincture and 70% alcohol. Second, local anesthesia was carried out with a paravertebral nerve block. Nerves thoracic (T) 13 and lumbar (L) 1, 2, and 3 were blocked with 10 ml 5% procaine (Sanma Animal Pharmaceutical Ltd., Harbin, China), and infiltration anesthesia was taken around the incision. Third, an ~20 to 25 cm vertical incision in the left para-lumbar fossa was made through blunt dissection of abdominal wall muscles and incision of the peritoneum. A 2 g sample of ruminal epithelia was cut from the underlying connective tissues in the ventral sac with surgical scissors.

To investigate the distribution of SLC5A8 in the gastrointestinal tract of cows and calves, three healthy adult cows (selected from control group cows randomly) and three calves were euthanized by exsanguination from the carotid artery following an intramuscular injection of 3 μL/kg of Xylazine hydrochloride (Huamu, Changchun, China), and 4 mL sodium heparin were injected in the jugular vein to avoid blood clotting. The gastrointestinal tract was removed from the abdominal cavity immediately after euthanasia, and samples of ~2 g were collected from the duodenum, jejunum, ileum, cecum, enlargement of the colon, rectum, ruminal ventral sac, reticulum, omasum, and abomasum, respectively.

The tissues were washed in ice-cold 0.9% NaCl (w/v) (pH 7.0) until no feed contents were visible in the tissue. For immunohistochemical studies, tissue samples were immediately fixed in 4% paraformaldehyde (BL539A; Biosharp, Hefei, China) for 24 h. After fixation, the tissues were dehydrated through a series of graded concentrations of ethanol and xylene, embedded in paraffin, serially sectioned into 4-μm slices, and mounted on poly-l-lysine-coated slides (16). For Western blotting and RT-PCR analyses, tissue samples were immediately frozen in liquid nitrogen and subsequently stored at −80°C until use (17).

### Collection, Isolation, and Culture of Primary Rumen Epithelial Cells

To isolate primary bovine ruminal epithelial cells, the rumen tissue was obtained through surgical excision from three calves mentioned above under sterile conditions (18). Serial trypsin (T8150; Solarbio, Beijing, China) digestions were performed to isolate ruminal epithelium cells as previously described (19, 20). Briefly, the forestomach of the calf, including the rumen, was taken out of the abdominal cavity within 2 to 3 min after euthanasia. The rumen was separated from the reticulum, omasum, and abomasum. The rumen samples were first washed three times in cold D-Hank's buffer and were then aseptically cut into small pieces (~4 cm^2^). Next, the minced laminae were washed two times (30 min each) with D-Hank's buffer containing penicillin (2,500 U/mL) and streptomycin (2,500 U/mL) (P1400; Solarbio, Beijing, China) and once with D-Hank's buffer containing amphotericin B (1,000 U/mL) (MB1014; Meilun, Dalian, China) and gentamicin (12 μg/mL) (Lishida, Beijing, China) for 30 min. Next, pieces of ruminal mucosa (0.25 cm^2^) were taken from the rumen sample using surgical instruments, including fine scissors and tweezers (Shanghai Medical Instruments Co., Ltd). Then, the ruminal tissue suspension was washed with PBS (pH 7.4; P1010; Solarbio) several times until the solution became muddy and was then centrifuged at 1,000 × rpm for 10 min at 4°C.

Primary ruminal cells were isolated from the mucosae using a digestion solution containing 0.25% trypsin and 0.02% EDTA-Na_2_ (E8030; Solarbio) for 15–20 min in a shaking warm-air bath at 37°C (21). The solution was filtered sequentially through 100-mesh (150-μm) sieves; the tissue remaining on the mesh was rinsed with 37°C D-Hank's buffer to remove any adhering cells, and the filtrate was rinsed and combined. Recovered cells from the initial digests were discarded because they consisted primarily of cells from stratum corneum and stratum granulosum layers, which are not representative of epithelial metabolism. Each fraction was filtered and examined under a phase contrast microscope. The tissues were placed into a flask with fresh 0.25% trypsin for another 15 min digestion. Cells were isolated using four to six cycles of digestion with fresh trypsin solution depending on the digestion status. Trypsinization was terminated after cell harvest by adding D-Hank's containing 15% fetal bovine serum (FBS) (FB15015; Clarkbio, Virginia, USA) into the digestion solution (at a ratio of 1:1, v/v). Then the epithelium cell suspension was washed twice with low-glucose Dulbecco's modified Eagle's medium (DMEM) (≤1 mg/mL glucose; HyClone, Logan, UT) and centrifuged at 1,000 × *rp*m (D-37520; ThermoFisher, Germany) for 10 min at 4°C to remove any residual trypsin from the pellets. After washing with low-glucose DMEM, the digest solution was also filtered through a 300 mesh (352340; Corning) into 50-ml polypropylene centrifuge tubes.

Finally, the ruminal epithelium cell yield was assessed using a hemocytometer (YA0811; Solarbio), and the cell viabilities were estimated using trypan blue dye (C0040; Solarbio); then, the ruminal epithelium cell density was adjusted to 1 × 10^6^ cells/mL with low-glucose DMEM. The ruminal epithelium cells were seeded into six-well tissue culture plates (2 mL per well) (3516; Corning) and incubated at 37°C in 5% CO_2_ in a humidified incubator (240i; Thermo Scientific). After 18 h, the medium was replaced with low-glucose DMEM containing 15% FBS, 200 U/mL penicillin, 200 U/mL streptomycin, 6 μg/mL gentamicin, and 6 μg/mL amphotericin B. The medium was replaced every 24 h.

### Immunohistochemistry of SLC5A8

To localize the SLC5A8 protein in tissue, 4-μm-thick tissue samples were used for antigen retrieval by heating for 15 min in a water bath kettle oven at 95°C in the presence of PBS (22). Samples were incubated in 3% (v/v) H_2_O_2_-methanol (M813895; Macklin Reagent, Shanghai, China) at room temperature for 10 min to quench endogenous peroxidase activity. Then, the sections were washed in PBS, followed by incubation with blocking buffer (normal goat serum) (SL038; Solarbio) at room temperature for 20 min to prevent non-specific reactions. Subsequently, sections were incubated overnight with the diluted anti-SLC5A8 (1:50; Cat. 21433-1-AP; Proteintech, Chicago, IL, USA) antibody at 4°C and were then washed with PBS, followed by incubation with HRP-goat anti-rabbit IgG (H + L) (ab6721, Abcam, Cambridge, UK) at a dilution of 1:200 for 30 min. The immunoreactivity of the sections were detected by treating with 0.5% 3,3′-diaminobenzidine tetrachloride in PBS containing 0.01% H_2_O_2_ and counterstained with hematoxylin (H810909; Macklin Reagent) to visualize the bound antibody. Negative control slides were incubated with PBS instead of primary antibody and included in each staining run.

### Immunocytofluorescence of SLC5A8

To determine the location of SLC5A8 protein in ruminal epithelium cells, cells were grown on glass coverslips to 90% confluency and were then fixed with 4% paraformaldehyde for 20 min at room temperature. EDTA-Na_2_ (1 m*M*) was used for antigen retrieval at 95°C for 5 min. Next, the ruminal epithelium cells were washed with PBS and exposed to the primary antibody SLC5A8 (diluted 1:100 with 0.1% PBS containing 5% goat serum) at 4°C overnight. Then the ruminal epithelium cells were incubated with goat anti-rabbit IgG conjugated with FITC (1:200; Beyotime Institute of Biotechnology, Shanghai, China) at room temperature for 30 min and counterstained with Hoechst 33258 (Beyotime) (23). Finally, the coverslips (YA0774; Solarbio) were sealed with glycerol (MB9893; Meilun), and the samples were observed by laser confocal microscopy (FV500, OLYMPUS, China) (24).

### Protein Extraction and Western Blotting of SLC5A8

Total proteins of ruminal epithelium cells and tissues were extracted using a protein extraction kit (Sangon Biotech Co. Ltd., Shanghai, China) according to the manufacturer's instructions. A BCA Protein Quantitation Kit (Beyotime) was used to evaluate the protein content. The protein samples (40 μg per lane) contained 4 × SDS sample buffer (P1016; Solarbio) and were denatured by boiling for 5 min. Then the protein components were separated on a 12% (w/v) sodium dodecyl sulfate-polyacrylamide gel electrophoresis gel and transferred to a polyvinylidene difluoride membrane (Millipore, Bedford, MA). Next, the membranes were blocked with 3% bovine serum albumin-Tris-buffered saline-Tween buffer (pH 7.4) for 4 h at room temperature and were then exposed to the primary antibodies SLC5A8 or β-actin (Proteintech, Chicago, IL, USA) at 4°C overnight. After being washed several times, the blocked membranes were incubated with a horseradish peroxidase (HRP)-conjugated secondary antibody (diluted 1:5000) (Boster Biological Technology Co. Ltd) at room temperature for 45 min. Finally, an imager (ProteinSimple, Santa Clara, CA, USA) was used to image the bands, which were visualized by enhanced chemiluminescence solution (ECL, Millipore). Protein intensity was quantified by the Gel-Pro Analyzer program normalized to β-actin levels (25). Each Western blot was performed a total of three times.

### RNA Extraction and Quantitative Real-Time PCR (qRT-PCR) of SLC5A8

Total RNA of ruminal epithelium cells and tissues were extracted with TRIzol reagent (TaKaRa Biotechnology Co. Ltd., Tokyo, Japan) according to the supplier's protocol. A K5500 MicroSpectrophotometer (Beijing Kaiao Technology Development Co. Ltd., Beijing, China) was used to determine the concentration of RNA before reverse transcription polymerase chain reaction (RT-PCR). Approximately 2 μg of total RNA was reverse-transcribed to cDNA in 20-μL reactions using PrimeScript Reverse Transcriptase (TaKaRa Biotechnology Co., Ltd.) according to the manufacturer's instructions. The mRNA expression levels were evaluated by using qRT-PCR with the SYBR Green QuantiTect RT-PCR Kit (TaKaRa) and a 7500 Real-Time PCR System (Applied Biosystems Inc.) (26). The relative expression of each target gene was determined by the 2^−ΔΔCT^ method. The amplification conditions were as follows: 95°C for 3 min, followed by 45 cycles of 95°C for 15 s and 60°C for 1 min. All the reactions were run in triplicate. The relative expression levels were normalized to actin β (*ACTB*) levels. The primer pairs for *SLC5A8* were 5′-GTGTGAACCAATCCCAAGTGC-3′ (forward) and 5′-AAGGATCACCCAGAGTCCCA-3′ (reverse). *ACTB* primers were 5′-GCCCTGAGGCTCTCTTCCA-3′ (forward), and 5′-GCGGATGTCGACGTCACA-3′ (reverse).

### SCFA Treatment of Primary Rumen Epithelial Cells

To detect the effect of SCFA on SLC5A8 expression, the composition and concentration of SCFA used in this study were chosen depending on the actual situation in the rumen of healthy and SARA dairy cows (27, 28). After being cultured in a sterile room for 7 days, the ruminal epithelium cells were treated with acetate (0, 30, 60, or 90 m*M*), propionate (0, 20, 30, or 40 m*M*), and butyrate (0, 10, 20, or 30 m*M*) (Sigma-Aldrich Co., St. Louis, MO), and to simulate the case-control groups, the rumen epithelial cells were treated with a combination of SCFA (control group: 60 m*M* acetate, 30 m*M* propionate, and 20 m*M* butyrate; SARA group: 90 m*M* acetate, 40 m*M* propionate, and 30 m*M* butyrate) for 6 h, respectively. The same concentration of NaCl (BL542A; Biosharp), simulating the concentration of SCFA treatment, was used to treat the rumen epithelial cells. Then, the pH was adjusted to 6.5 by the addition of 1 *M* NaOH (Sangon) dropwise over a period of 15 to 20 min (29, 30). Stock SCFA solution was prepared as follows: 8.203 g of acetate, 9.606 g of propionate and 11.009 g of butyrate was dissolved in 100 mL of distilled water and sterilized by filtration to obtain concentration of 1 *M* for each acid, and stored at −20°C.

To measure the viability of rumen epithelial cells in response to SCFA treatment a 3-(4,5)-dimethylthiahiazo(-z-y1)-3,5-di-phenytetrazoliumromide (MTT) colorimetric assay was done according to the manufacturer's instructions (Beyotime). Culture medium was carefully removed and exchanged with fresh media. MTT solution (5 mg/mL PBS) was then added, and the plate was placed in an optimal atmosphere at 37°C.

### Statistical Analysis

Data analysis was conducted in SPSS 19.0 software (IBM, Chicago, IL). All statistical tests were two-sided. Significance was declared at *P* ≤ 0.05 and a tendency at 0.05 to 0.10. The results are presented as the least squares mean ± standard error. When we had single data of animals, such as for ruminal SCFA concentrations and SLC5A8 expression data of healthy, control, and SARA cows, we used an unpaired *t-* test assuming equal distribution. When we had pre–post data of animals, such as for ruminal SCFA concentrations and SLC5A8 expression data of SARA cows before and after treatment, we used a paired *t*-test. Multiple data, such as the SLC5A8 distribution data and the SCFA dosage study of the same animal, were analyzed as a repeated-measures-in-time ANOVA study.

## Results and Discussion

The objective of this study was to evaluate the potential role of SLC5A8, a low-affinity/high-capacity SCFA^−^/bicarbonate antiporter (11) in the etiology of bovine SARA. At physiological pH (between 6 and 7 in ruminal and intestinal contents), SCFA (pKa of SCFA ~4.8) are present as anions in the bovine gastrointestinal tract, which limits their passive diffusion across the absorptive membrane; thus, SCFA are primarily absorbed via facilitated transmembrane transport mechanisms (31). However, our understanding of bovine SCFA transport mechanisms is limited. The membrane transport protein SLC5A8, using a Na^+^-coupled transport system, plays a major role in the absorption of SCFA in the gastrointestinal tract of mouse, rat, and humans (32, 33); however, the role of SLC5A8 in bovine is unknown. Given the highly conserved protein sequence of bos taurus SLC5A8 (NP_001158333.1) between homo sapiens (NP_666018.3, 89.2%), mus musculus (NP_663398.2, 86.58%), and ovis aries (XP_004006709.1, 98.36%) (from NCBI), we hypothesized that SLC5A8 may play an important role in bovine SCFA absorption and in the etiology of SARA.

### Distribution and Localization of SLC5A8

**Figure 1:**
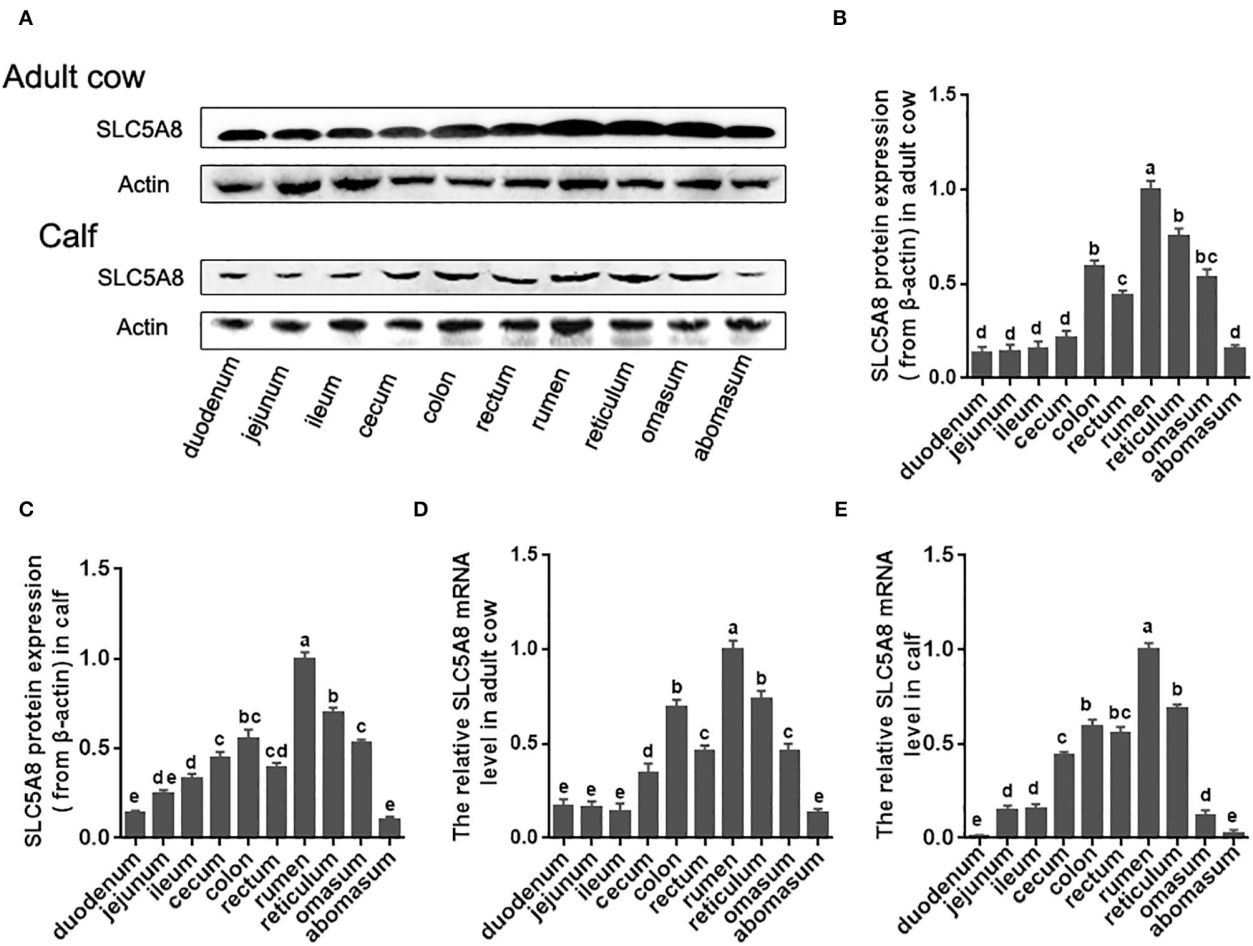
Protein and gene expression of SLC5A8 along the gastrointestinal tract of calves and adult cows. **(A)** Representative Western blot of SLC5A8 along gastrointestinal tract of an adult dairy cow and 1-day-old calf. **(B)** Relative protein expression of SLC5A8 (after adjusting for β-actin) of adult dairy cows **(C)** and 1-day-old calves. **(D)** Relative mRNA expression of *SLC5A8* (after adjusting for *ACTB*) of adult dairy cows **(E)** and 1-day-old calves. Data in **(B–E)** are presented as mean ± SEM (*n* = 3). Letters indicate differences at *P* < 0.05.

**Figure 2:**
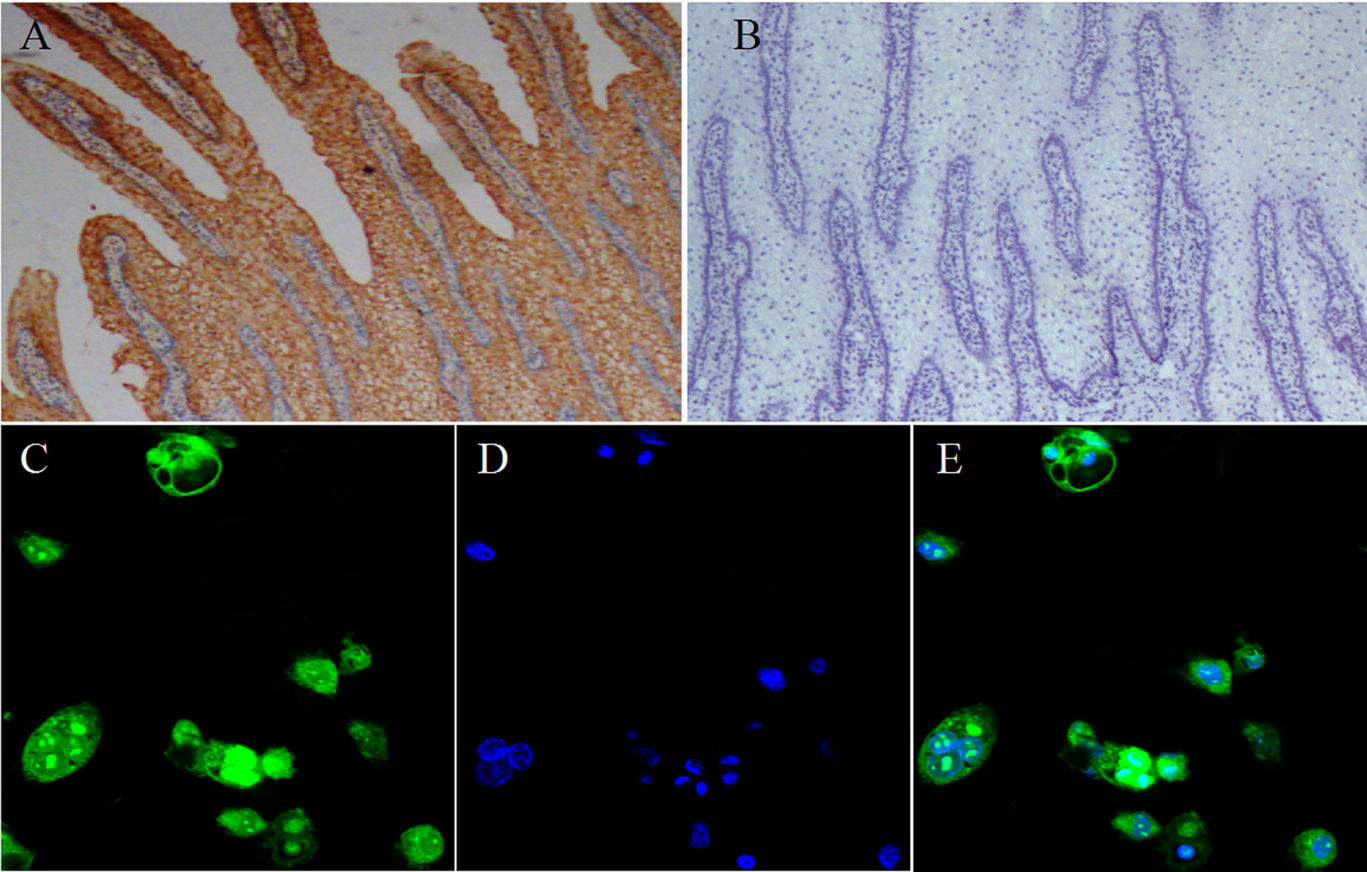
Localization of SLC5A8 in ruminal epithelial cells. Representative immunohistochemical staining of ruminal tissue (40× magnification) with a SLC5A8 antibody **(A)** SLC5A8-positive cells are stained brown or its negative control **(B)** ruminal epithelia incubated with antigen-bound SLC5A8 antibody (Magnifications: 40×). **(C)** Representative immunofluorescence staining of ruminal epithelial cells (400 × magnification) for SLC5A8, **(D)** DNA using Hoechst 33258 staining, and **(E)** superimposed image of **(C,D)**.

To test our hypothesis, we first investigated the currently unknown SLC5A8 gene and protein expression patterns in various parts of the bovine gastrointestinal tract (Figure 1). Protein and mRNA expression of SLC5A8 was detected throughout the gastrointestinal tract in adult cows and calves and followed similar trends. In line with bovine SCFA absorption patterns, the highest expression levels were in the forestomachs, being most prominent in the rumen; intermediate levels were in the large intestine; and low levels were in the abomasum and small intestine. Within the rumen, SLC5A8 was primarily localized in the rumen epithelium of the proximal chamber (Figures 2A,B), the site of primary SCFA absorption (33). Within the rumen epithelium, SLC5A8 was primarily localized at the membranes of the ruminal epithelial cells (Figures 2C–E) as would be expected from a membrane transport protein (32). In summary, the distribution and localization of SLC5A8 across the gastrointestinal tract supports a potential role in bovine SCFA absorption. Furthermore, our previous study also showed that the presence of proton-linked monocarboxylate transporter (MCT1), a transportation protein for the SCFA, along the gastrointestinal tract of cows and most abundantly expressed in the rumen epithelium (16). This further suggests that these transportation proteins play a vital role in the transportation of SCFA in ruminants.

### Effect of SCFA on SLC5A8 Expression

**Figure 3:**
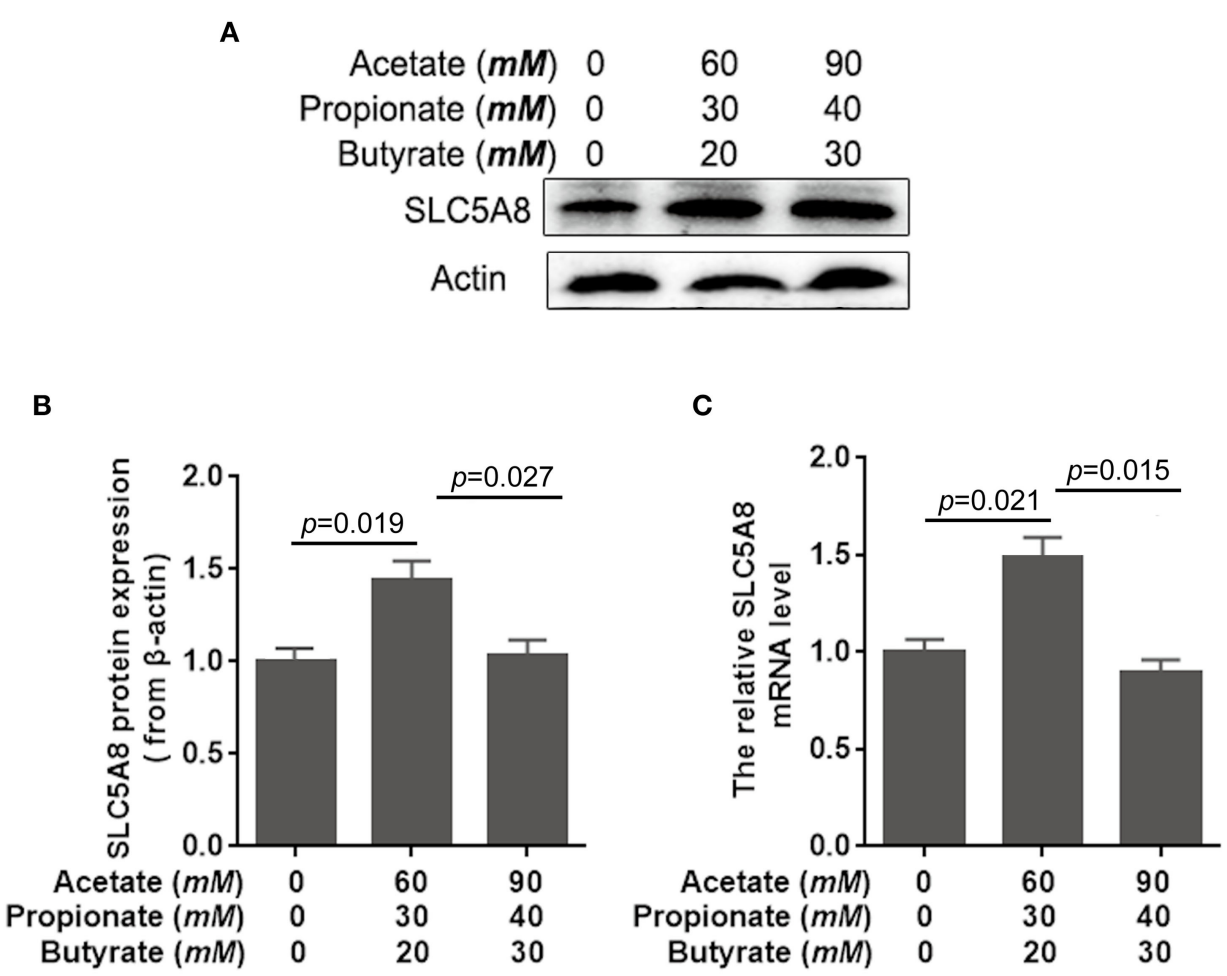
Effects of short-chain fatty acids on mRNA and protein expression of SLC5A8 in rumen epithelium cells. The cells of 1-day-old calves were treated with either no acetate, propionate, and butyrate (zero-control), 60 m*M* acetate, 30 m*M* propionate, and 20 m*M* butyrate (typical of healthy cows; Table 3), and 90 m*M* acetate, 40 m*M* propionate, and 30 m*M* butyrate (typical for cows with SARA, Table 3). **(A)** Western blotting results of SLC5A8 and β-actin in ruminal epithelium cells. **(B)** Relative grayscale of SLC5A8. **(C)** The relative mRNA levels of *SLC5A8* in ruminal epithelium cells. The data presented are the mean ± SEM.

Rapid ruminal fermentation of carbohydrates leads to SCFA accumulation in the rumen as SCFA production surpasses facilitated SCFA absorption (34, 35). Currently unknown is how SCFA accumulation alters facilitated SCFA absorption. Addition of SCFA at concentrations typical for healthy cows (60 m*M* acetate, 30 m*M* propionate, and 20 m*M* butyrate; Table 3) increased similarly SLC5A8 protein expression (+43.8%; *P* = 0.019; Figures 3A,B) and gene expression (+49.5%; *P* = 0.021; Figure 3C) in rumen epithelial cells. Accumulation of SCFA beyond physiological concentrations as observed in cows with SARA (90 m*M* acetate, 40 m*M* propionate, and 30 m*M* butyrate; Table 3) decreased similarly SLC5A8 protein expression (−28.5%; *P* = 0.027; Figures 3A,B) and gene expression (−40.4%; *P* = 0.015; Figure 3C) back to levels without SCFA addition. Thus, SCFA accumulation may limit SLC5A8-facilitated SCFA absorption in the rumen.

**Table 3:** Rumen concentration of short-chain fatty acids (SCFA) in healthy cows and cows with subacute ruminal acidosis (SARA).

**Ruminal parameters**	**Control**	**SARA**	***P*-value**
	***N* = 5**	***N* = 5**	
Total SCFA, m*M*	107.58 ± 8.07	174.05 ± 10.09	0.005
Acetate, m*M*	60.12 ± 5.40	95.13 ± 6.48	0.033
Propionate, m*M*	27.28 ± 1.61	41.36 ± 2.37	0.009
Acetate: Propionate	2.31 ± 0.13	2.03 ± 0.16	0.012
Butyrate, m*M*	19.66 ± 1.03	29.85 ± 1.94	0.032
Isobutyrate, m*M*	2.47 ± 0.07	3.73 ± 0.11	0.004
Lactate, m*M*	1.35 ± 0.05	2.98 ± 0.10	0.001

*All data represent the mean ± SEM*.

**Figure 4:**
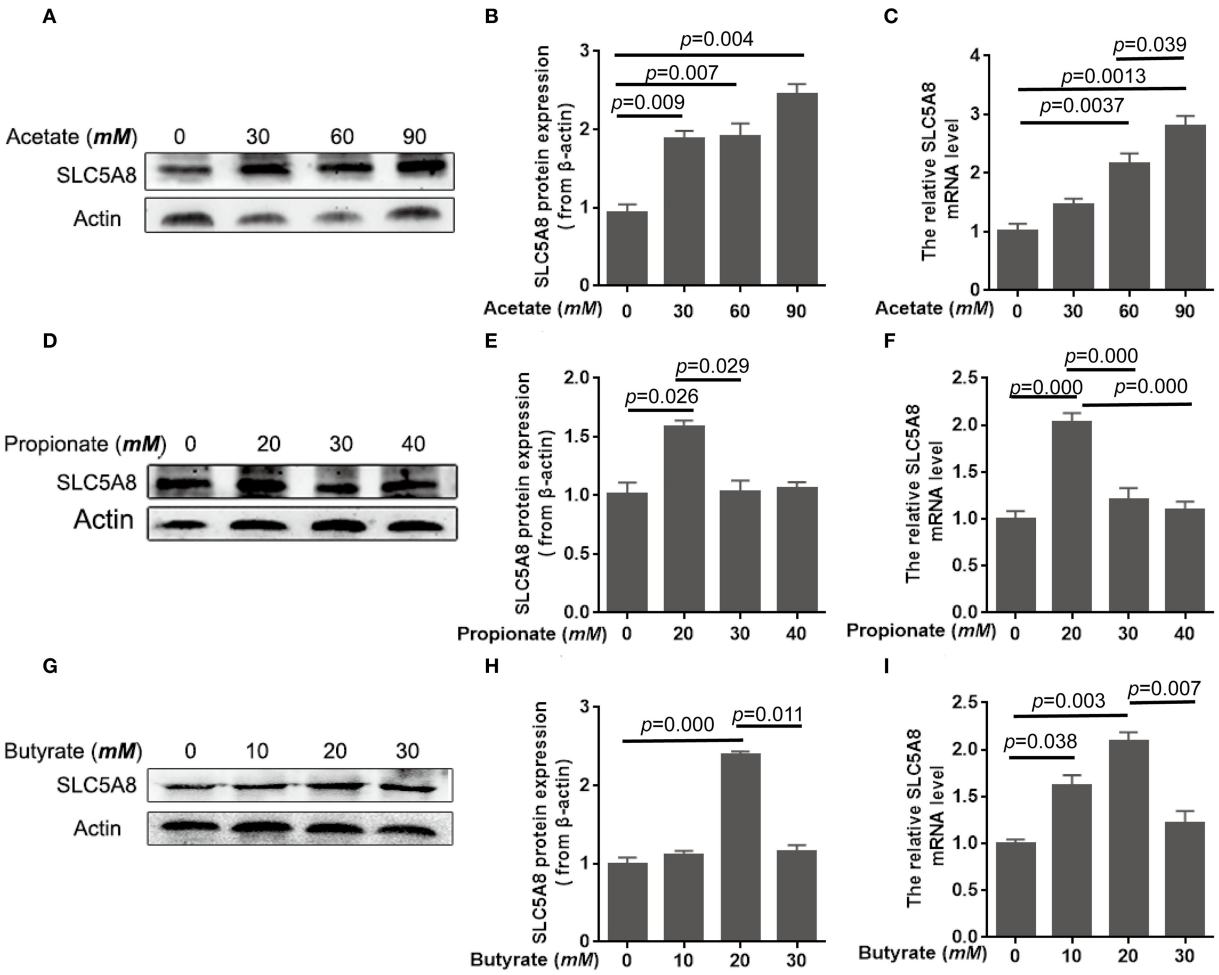
Effects of acetate, propionate, and butyrate on mRNA and protein expression of SLC5A8 in rumen epithelium cells. The cells were treated with acetate (0, 30, 60, or 90 m*M*; **A–C**), propionate (0, 20, 30, or 40 m*M*; **D–F**), or butyrate (0, 10, 20, or 30 m*M*; **G–I**). **(A,D,G)** Western blotting results of SLC5A8 and β-actin. **(B,E,H)** Relative grayscale of SLC5A8. **(C,F,I)** The relative mRNA levels of *SLC5A8*. The data presented are the mean ± SEM.

**Figure 5:**
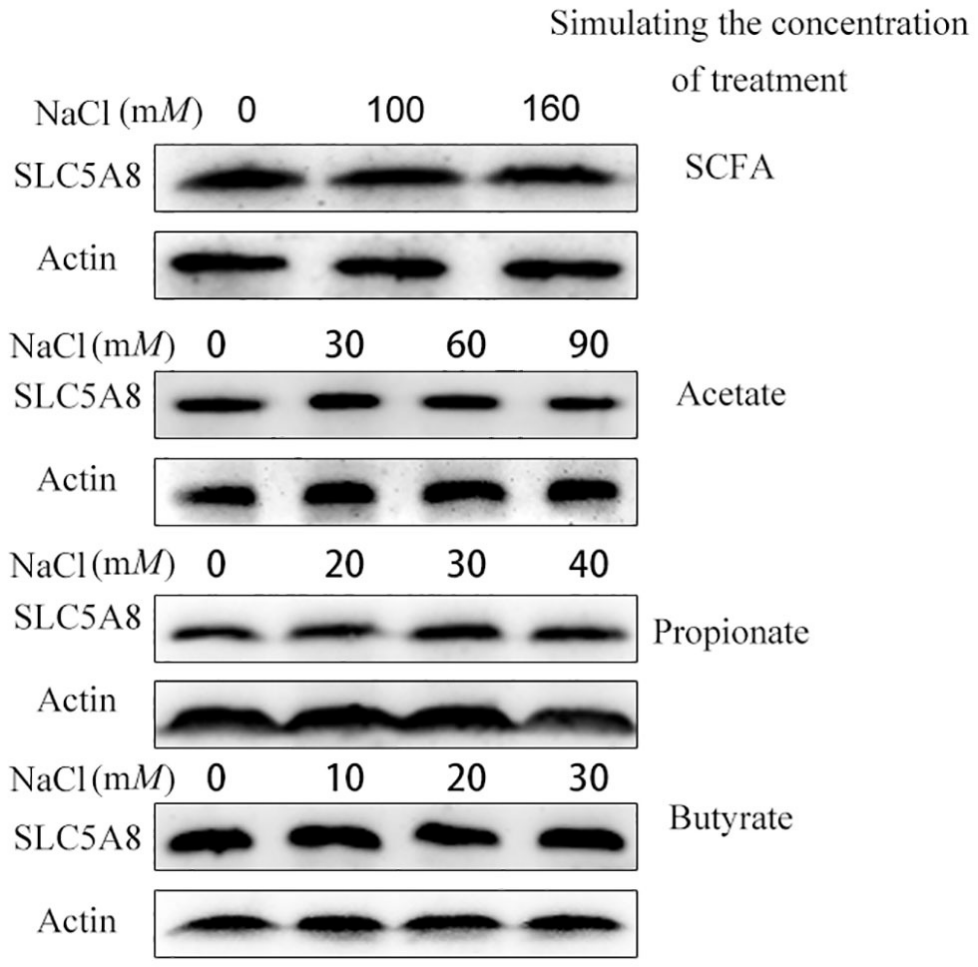
Effect of different osmotic pressure on the mRNA and protein expression of SLC5A8 in rumen epithelium cells. The cells were treated NaCl (0, 100, or 160 m*M*), NaCl (0, 30, 60, or 90 m*M*), NaCl (0, 20, 30, or 40 m*M*), and NaCl (0, 10, 20, or 30 m*M*). Western blotting results of SLC5A8 and β-actin.

**Figure 6:**
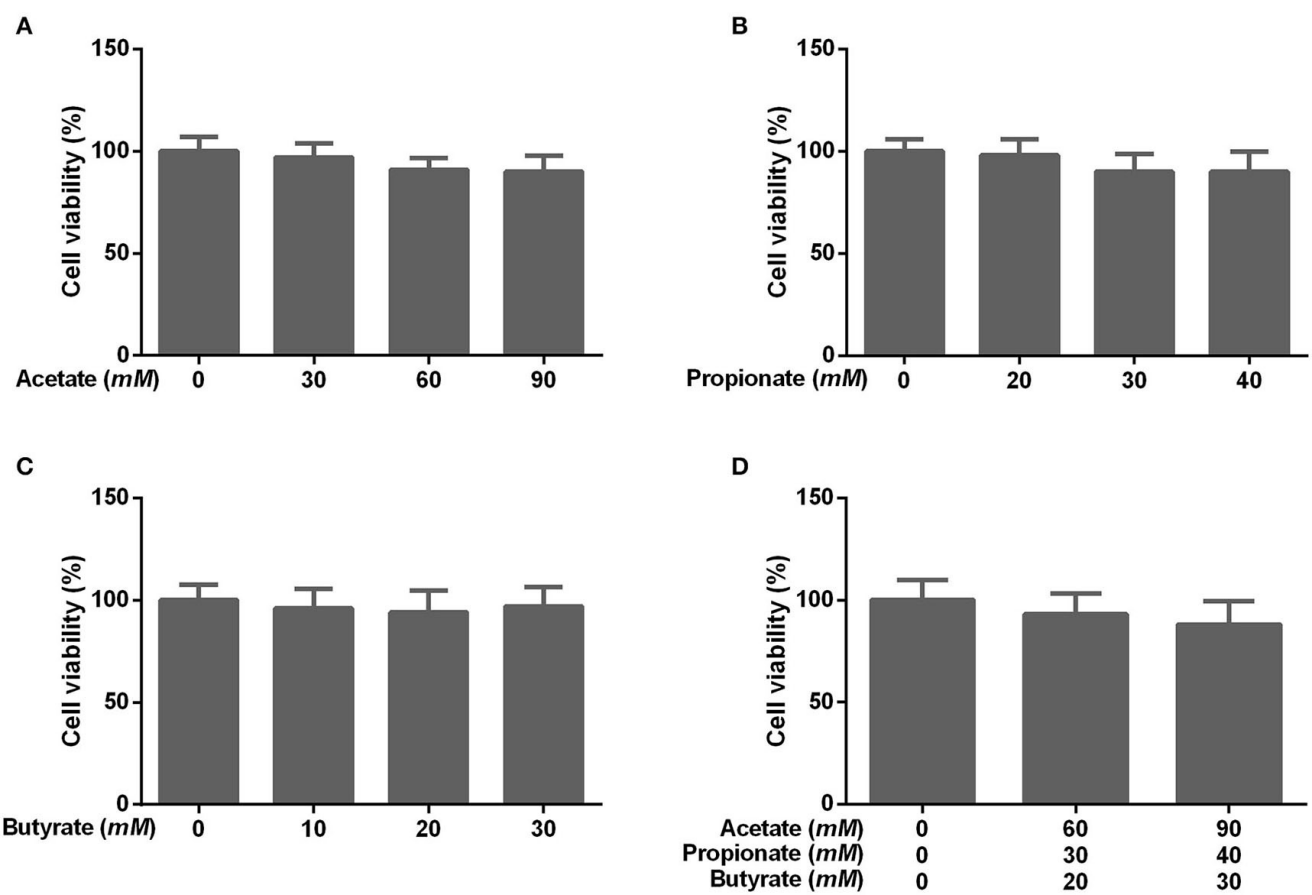
Effects of different concentrations of acetate, propionate, butyrate, and SCFA on rumen epithelium cell viability. The cells were treated with **(A)** acetate (0, 30, 60, or 90 m*M*), **(B)** propionate (0, 20, 30, or 40 m*M*), **(C)** butyrate (0, 10, 20, or 30 m*M*), or **(D)** no acetate, propionate, and butyrate (zero-control), 60 m*M* acetate, 30 m*M* propionate, and 20 m*M* butyrate (typical of healthy cows; Table 3), and 90 m*M* acetate, 40 m*M* propionate, and 30 m*M* butyrate (typical for cows with SARA, Table 3). The data presented are the mean ± SEM.

To determine the effect of individual SCFA on SLC5A8 expression in rumen epithelial cells, a dosage experiment at physiological SCFA concentrations was conducted. Acetate addition between 0 and 90 m*M* linearly increased protein and gene expression of SLC5A8 (Figures 4A–C); similarly, acetate transport in sheep omasum increased linearly with luminal acetate concentrations (36). In contrast, addition of propionate (Figures 4D–F) and butyrate (Figures 4G–I) followed a similar pattern as total SCFA addition (Figures 3A,B) with the highest SLC5A8 expression at 20 m*M* of propionate and butyrate, respectively, concentrations typical for healthy cows (Table 3). To eliminate NaCl concentrations, used as a control for different osmolarity, as potential reason for the SLC5A8 expression pattern, we determined the effect of NaCl concentration on SLC5A8 expression and observed no significant group differences (Figure 5). To eliminate cell viability as potential reason for the SLC5A8 expression pattern, we determined the effect of individual and total SCFA addition on SLC5A8 expression and observed no significant group differences (Figure 6). Thus, accumulation of propionate and butyrate may limit SLC5A8-facilitated SCFA absorption in the rumen. Furthermore, SLC5A8 was reported as a transporter of lactate in mice and human (32, 33). In SARA, lactate is an important factor to induce the decrease of the ruminal pH. The results showed that the concentration of lactate increased in the rumen of SARA cows (Table 3) and decreased after treatment (Table 4). However, the effect of lactate on SLC5A8 expression was not measured in this study, which is a limitation for this study.

**Table 4:** Rumen concentration of short-chain fatty acids (SCFA) before and after therapeutic treatment of cows with subacute ruminal acidosis (SARA).

**Ruminal parameters**	**Before**	**After**	***P*-value**
	***N* = 5**	***N* = 5**	
Total SCFA, m*M*	174.05 ± 10.09	113.23 ± 8.73	0.003
Acetate, m*M*	95.13 ± 6.48	67.48 ± 4.53	0.040
Propionate, m*M*	41.36 ± 2.37	28.66 ± 1.57	0.008
Acetate: Propionate	2.03 ± 0.16	2.28 ± 0.17	0.014
Butyrate, m*M*	29.85 ± 1.94	21.87 ± 0.88	0.050
Isobutyrate, m*M*	3.73 ± 0.11	2.89 ± 0.08	0.010
Lactate, m*M*	2.98 ± 0.10	1.52 ± 0.11	0.001

*All data represent the mean ± SEM*.

### Effect of SARA on SLC5A8 Expression

**Figure 7:**
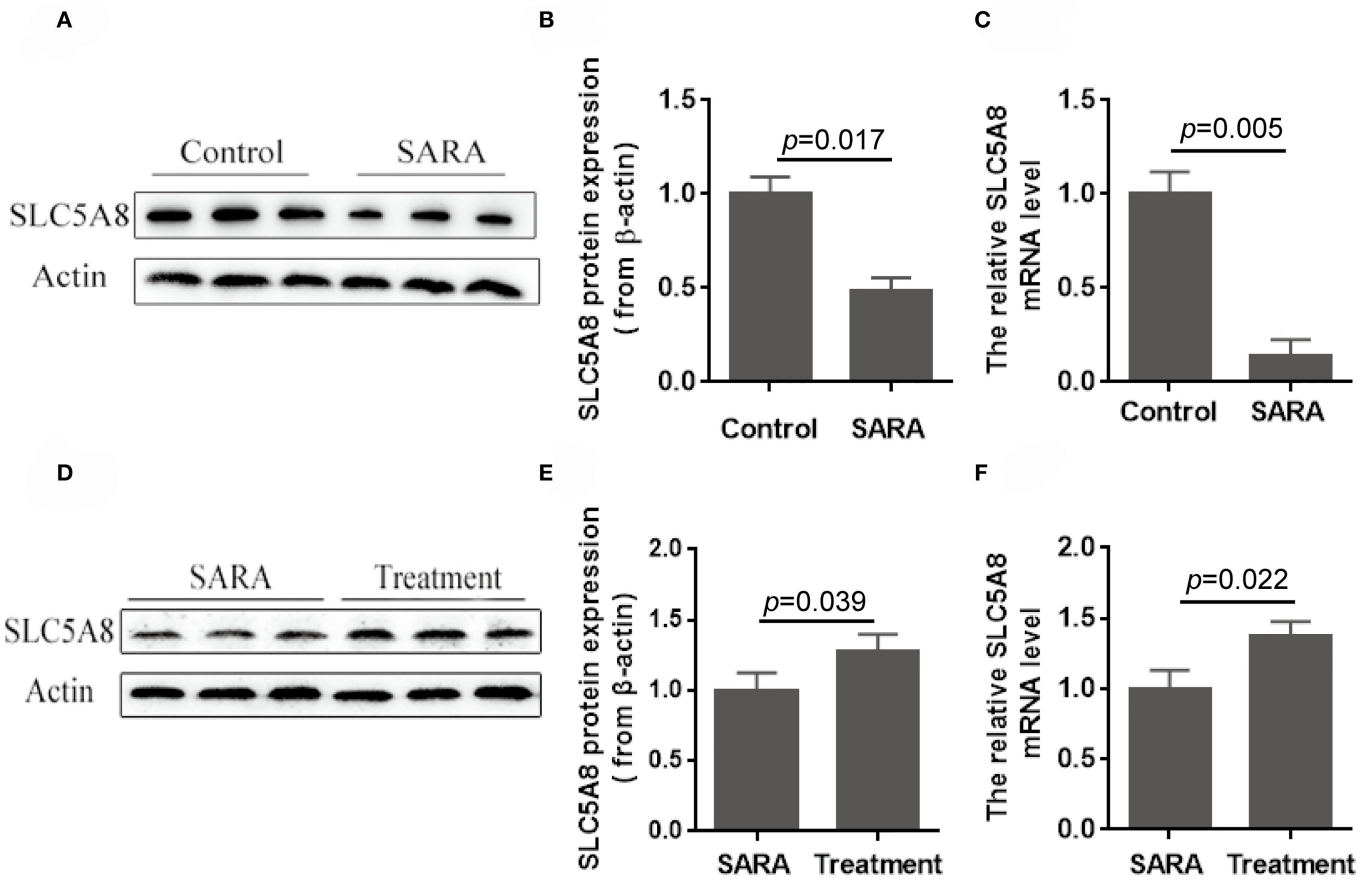
Protein and gene expression of SLC5A8 in healthy control cows and cows with SARA before and after SARA therapeutic treatment. **(A)** Representative Western blots of SLC5A8 in the rumen of healthy control and SARA cows. Relative protein **(B)** and mRNA **(C)** expression of SLC5A8 (after adjusting for β-actin) in the rumen of healthy control and SARA cows. **(D)** Representative Western blots of SLC5A8 in the rumen of SARA cows before and after successful treatment of SARA. Relative protein **(E)** and mRNA **(F)** expression of SLC5A8 (after adjusting for β-actin) in the rumen of SARA cows before and after successful treatment of SARA. Data in **(B,C,E,F)** are presented as mean ± SEM (*n* = 5).

Accumulation of SCFA in the rumen (8, 9) and the resulting low rumen pH are hallmarks of SARA, commonly defined by rumen fluid pH < 5.6 for at least 3–5 h over a 24-h period (1). Currently unknown is whether the low pH is due to greater SCFA production, decreased SCFA absorption across the rumen epithelium, or both. Compared with healthy control cows, SARA cows had higher concentrations of SCFA in the rumen (Table 3) and lower protein expression (−50.2%; *P* = 0.017; Figures 7A,B) and gene expression (−86.1%; *P* = 0.005; Figure 7C) of SLC5A8 in rumen epithelium. Treatment of SARA decreased SCFA concentrations in the rumen (−34.9%; *P* = 0.003; Table 4) and increased protein expression (+28.2%; *P* = 0.039; Figures 7D,E) and gene expression (+36.8%; *P* = 0.022; Figure 7F) of SLC5A8 in rumen epithelium; however, the expression levels were still lower than in healthy control cows. Thus, decreased expression of SLC5A8 in the rumen may play a role in the etiology of SARA. Some clinical signs, such as intermittent diarrhea, dehydration, depression, decreased rumen motility, and laminitis, were improved after SARA treatment, but milk volume, body condition, and body weight were not significantly changed.

A limitation of this study is that we were not able to use functional measures of SLC5A8-faciliated SCFA absorption as gene and protein expression patterns may or may not translate into functional changes. Importantly, the absorption rates of short-chain fatty acids are closely correlated with the gene and protein expression of SLC5A8 in non-ruminants. Miyauchi reported that uptake of SCFA was higher in *SLC5A8* cRNA-injected oocytes from Xenopus laevis than in uninjected oocytes (32). The similarities of gene and protein expression patterns of SLC5A8 led us to conclude that SCFA may alter SLC5A8 expression via epithelial cell surface receptors on the transcriptional level. It cannot be excluded that SARA cows have low-functioning SLC5A8 transporters. Given the low number of cows from a single farm (*n* = 5 per group), validation of the results with various SARA models are warranted. Furthermore, this study cannot prove cause and effect as we did not overexpress SLC5A8 in SARA cows to treat SARA or decrease SLC5A8 in healthy cows to cause SARA. Besides SLC5A8, many other transport proteins are involved in facilitated SCFA absorption, such as MCT1-4 (*SLC16A1-4*), DRA (*SLC26A3*), PAT1 (*SLC26A6*), and AE2 (*SLC4A2*) (37, 38). The expression changes of these transport systems are associated with the SCFA accumulation and development of SARA. Compared with other transport protein, SLC5A8 is widely characterized in non-ruminants. In this study, we investigate the expression distribution and changes of SLC5A8 in SARA cows and the effect of SCFA on SLC5A8 expression in rumen epithelial cells. Given the role of other transport systems on SCFA absorption, we will examine in future studies the impact of other membrane transport proteins on SCFA accumulation and SARA.

In summary, accumulation of propionate and butyrate decreases protein and gene expression of SLC5A8 in rumen epithelium, a membrane protein important for SCFA absorption. Gene and protein expression of SLC5A8 can be partly restored by decreasing SCFA accumulation in cows with SARA. Future studies are warranted to confirm the potential role of SLC5A8 in rumen SCFA accumulation and in the etiology and therapy of SARA. Moreover, exploration of the control of SLC5A8 gene may offer novel options for the prevention and therapy of SARA in dairy cows.

## Data Availability Statement

All datasets generated for this study are included in the article/supplementary material.

## Ethics Statement

The animal study was reviewed and approved by Animal Care and Use Committee of Jilin University.

## Author Contributions

CZ and GL conceived the study and participated in its design. CZ and YW performed the experimental work and wrote the manuscript. XZ, ZZ, and SZ assisted in the phenotyping and cytokine analysis. GS, XY, and XL collected the sample and the data. GB and GL participated in the writing of the manuscript and its critical review. All co-authors revised the manuscript and approved the final submitted version.

## Conflict of Interest

The authors declare that the research was conducted in the absence of any commercial or financial relationships that could be construed as a potential conflict of interest.
